# Inhibitory effects of *Δ*8-tetrahydrocannabinol on major hepatic cytochrome P450 enzymes and implications for drug disposition

**DOI:** 10.1016/j.dmd.2025.100122

**Published:** 2025-07-16

**Authors:** Mengqi Zhao, Shelby Coates, Keti Bardhi, Philip Lazarus

**Affiliations:** 1 Division of Molecular Biosciences, Department of Pharmaceutical Sciences, School of Pharmacy and Pharmaceutical Sciences, University at Buffalo, Buffalo, New York; 2 Department of Pharmaceutical Sciences, College of Pharmacy and Pharmaceutical Sciences, Washington State University, Spokane, Washington

**Keywords:** Cannabinoid, Cannabis, CYP450, Drug-drug interactions, Tetrahydrocannabinol

## Abstract

The increased use of cannabis in many parts of the United States and other countries has led to a need for a more comprehensive understanding of cannabis constituents and their potential for drug-drug interactions. *Δ*8-Tetrahydrocannabinol (*Δ*8-THC) is a psychoactive cannabinoid that is found at low concentrations in cannabis but is growing in popularity, especially where the use of *Δ*9-THC is restricted. Although certain cannabinoids including cannabidiol (CBD) are known to inhibit several metabolizing enzymes including many in the cytochrome P450 family, the effects of *Δ*8-THC remain poorly characterized. This study evaluated the inhibitory potential of *Δ*8-THC and its metabolites, 11-hydroxy-*Δ*8-THC and 11-nor-*Δ*8-THC-9-carboxylic acid, on major hepatic cytochrome P450 enzymes using in vitro assays with recombinant P450-overexpressing microsomes and pooled human liver microsomes. *Δ*8-THC and 11-hydroxy-*Δ*8-THC significantly inhibited CYP2C9- and CYP3A4-mediated metabolism in a dose-dependent, reversible manner. Lineweaver–Burk analysis indicated competitive inhibition for CYP2C9-mediated warfarin hydroxylation and noncompetitive inhibition for CYP2C9- and CYP3A4-mediated metabolism of diclofenac and midazolam, respectively. In contrast, 11-nor-*Δ*8-THC-9-carboxylic acid showed no significant inhibition of P450 enzymes. Static modeling predicted clinically relevant drug interactions, particularly with oral *Δ*8-THC. These findings underscore the potential for *Δ*8-THC to impact the pharmacokinetics of coadministered drugs and highlight the need for further clinical studies.

**Significance Statement:**

This study is the first to assess how *Δ*8-tetrahydrocannabinol and its active metabolites inhibit key hepatic P450 enzymes. Results suggest a high risk of *Δ*8-tetrahydrocannabinol–related drug interactions, especially with oral use, underscoring the need for clinical caution and further research.

## 1 Introduction

Cannabis, one of the most widely used natural products worldwide, has been valued for centuries for its medicinal and recreational properties. More than 100 different cannabinoids have been identified in cannabis, with cannabidiol (CBD) and *Δ*9-tetrahydrocannabinol (*Δ*9-THC) among the most extensively studied. CBD is known for its low psychoactive properties and therapeutic benefits, including anxiety relief and anti-inflammatory effects.^1^ In contrast, *Δ*9-THC is the main psychoactive component responsible for the "high" associated with cannabis use.^2^ Their popularity stems from a growing interest in natural health products, shifting legal landscapes, and strong evidence supporting their potential to treat a range of conditions—from chronic pain to epilepsy.^3^ As research expands, cannabinoids continue to drive public attention and scientific exploration, shaping how cannabis is understood and used worldwide.

Although not usually abundant in cannabis, *Δ*8-THC exhibits similar but milder psychoactive properties than *Δ*9-THC with reduced anxiety and sedation,^4^ and it has surged in popularity especially in regions where *Δ*9-THC is restricted.^5^ Structurally similar to *Δ*9-THC, *Δ*8-THC differs only in the location of a double bond, lying on the eighth carbon of the THC molecule as compared to the ninth carbon on *Δ*9-THC.^4^ Its increasing availability is largely due to legal ambiguities in cannabis regulation. *Δ*8-THC can be derived from hemp-derived CBD and it is less legally restricted than *Δ*9-THC.^6^ This has led to a flood of *Δ*8-THC products including edibles, tinctures, and vape cartridges into the consumer market.^7^ A March 2024 survey found that approximately 11% of US 12th graders had used *Δ*8-THC in the past year.^8^ Some states, including Alaska, Arizona, Arkansas, and Colorado, have banned *Δ*8-THC, whereas others lack specific legislation. The rapid rise in use raises pressing concerns about its public health implications, particularly its potential to interact with other medications.^9^

The liver plays a central role in drug metabolism, largely through the action of the cytochrome P450 (P450) enzyme system.^10^ P450 enzymes are responsible for the metabolism of most Food and Drug Administration-approved drugs, with enzymes like CYPs 1A2, 2C9, 2C19, 2D6, and 3A4 being especially significant.^11^ Any substance that inhibits or induces these enzymes can affect how other drugs are processed, potentially altering their efficacy or toxicity.^12^ Both *Δ*9-THC and CBD are metabolized by CYPs 2C9, 2C19, and 3A4, and studies show that they can inhibit multiple P450 enzymes. For example, CBD is a potent inhibitor of CYPs 2D6, 2C9, 3A4, and 2C19, which affects the metabolism of medications like dextromethorphan, warfarin, midazolam, and omeprazole.^13^ *Δ*9-THC also inhibits CYPs 1A2 and 2C9, affecting the metabolism of drugs such as phenacetin and diclofenac.^14^ The degree of interaction depends on dosage, frequency, and individual metabolic differences, highlighting the clinical importance of cannabinoid-drug interactions.^3^

Given its structural similarity to *Δ*9-THC, *Δ*8-THC likely possesses comparable pharmacological properties. For example, it acts as a partial agonist of CB1 and CB2 receptors, with approximately half the potency of *Δ*9-THC.^15^ Similar to that observed for *Δ*9-THC, once consumed, *Δ*8-THC is primarily metabolized to its active metabolite 11-hydroxy-*Δ*-8-THC (11-OH-*Δ*8-THC) by CYP2C9,^16^ then oxidized to 11-nor-*Δ*8-tetrahydrocannabinol-9-carboxylic acid (*Δ*8-THC-COOH) and excreted in the urine.^17^ Inhalation leads to rapid absorption, with peak plasma concentrations within 10 – 30 minutes. Oral consumption results in slower absorption, with peak effects occurring in 1 – 2 hours.^18^

Despite these biochemical insights, direct studies on the impact of *Δ*8-THC on P450 enzymes have not yet been performed. In the present study, the effects of *Δ*8-THC on the activities of several major hepatic P450 enzymes were examined. Results from the present studies indicate a strong potential for drug-drug interactions (DDIs), underscoring the need for caution among individuals using *Δ*8-THC alongside prescription medications and calling for further research into its clinical interactions.

## 2 Material and methods

### 2.1 Chemicals and reagents

*Δ*8-THC, 11-OH-*Δ*8-THC, and *Δ*8-THC-COOH were obtained from Cayman Chemical. Pooled human liver microsomes (HLMs; mixed gender, *n* = 50 donors) were purchased from Sekisui Xenotech. An NADPH-regenerating system was acquired from Corning. P450-specific probe substrates (phenacetin, bupropion, warfarin sodium, amodiaquine, diclofenac, omeprazole, dextromethorphan, chlorzoxazone, testosterone, and midazolam) of tested P450 enzymes were acquired from LGC Standards, as were their corresponding metabolite standards and internal standards (alpha-hydroxy midazolam-d4, 4-hydroxydiclofenac-d5, and (S)-7-hydroxywarfarin-d5). Liquid chromatography-mass spectrometry (LC-MS)-grade solvents, microcentrifuge tubes, and BCA assay kits were obtained from Fisher Scientific. All other chemicals were analytical grade or higher. The anti-V5 tag monoclonal antibody horseradish peroxidase (HRP) (Catalog No. R96125) was purchased from Novex, Fisher Scientific, whereas the anti-calnexin polyclonal rabbit antibody (Catalog No. 2433) and the anti-rabbit IgG, HRP-linked antibody (Catalog No. 7044S) were obtained from Cell Signaling Technology.

### 2.2 Cytochrome P450 enzyme inhibition assays

V5-tagged human P450 enzymes (CYPs 1A2, 2B6, 2C8, 2C9, 2C19, 2D6, 2E1, and 3A4) were cloned and overexpressed in HEK293 cells and microsomal fractions were isolated by differential centrifugation as previously described,^19^ with expression monitored by western blot analysis. An anti-V5-tagged antibody (1:1000) and an anti-calnexin polyclonal rabbit antibody (1:1000) together with an anti-rabbit IgG, HRP-linked antibody (1:5000) were used to probe V5 and calnexin, respectively (Supplemental Fig. 1). Briefly, microsomes (25 *μ*g) were electrophoresed on a 10% SDS–polyacrylamide gel and proteins were transferred to polyvinylidene difluoride membranes using a iBlot Gel Transfer Stacks polyvinylidene difluoride, Regular kit (Catalog No. IB401001). Membranes were blocked with 5% nonfat milk and incubated overnight at 4°C with a polyclonal rabbit anti-calnexin antibody (1:1000 dilution). They were then incubated for 1 hour at room temperature with an HRP-linked anti-rabbit IgG antibody, followed by incubation with a monoclonal anti-V5 tag antibody. Protein bands were visualized using the SuperSignal Femto Maximum Sensitivity Substrate (Bio-Rad). Calnexin bands served as the loading control for microsomal protein samples, whereas V5-HRP bands indicated the expression of each P450 enzyme, which was not detected in microsomes from parental HEK293 cells. Microsomal protein quantified using the BCA protein assay following the manufacturer’s instructions. To assess P450 enzyme inhibition, *Δ*8-THC, 11-OH-*Δ*8-THC, and *Δ*8-THC-COOH (1 or 10 *μ*M) were incubated with probe substrates in a reaction mixture containing 3 mM MgCl_2_, potassium phosphate buffer (pH 7.4), and either 20 – 50 *μ*g of recombinant CYP microsomes or 20 *μ*g of pooled HLM. Reactions were preincubated at 37°C for 3 minutes before initiating with an NADPH-regenerating system. Incubation times were 5 – 30 minutes for recombinant P450 microsomes (Supplemental Table 1) and 10 minutes for HLM. Reactions were terminated by the addition of 30 *μ*L of ice-cold acetonitrile, followed by vortexing and centrifugation at 17,000*g* for 15 minutes. Approximately 30 *μ*L of the supernatant was transferred to an ultraperformance liquid chromatography (UPLC) vial for analysis. To reduce nonspecific binding of cannabinoids, all incubations were performed using low-binding 0.6-mL tubes. Probe substrates were used at concentrations near their Michaelis-Menten constants ($K_{m}$) to minimize off-target interactions.^13^^,^^20^, ^21^, ^22^, ^23^, ^24^, ^25^

### 2.3 Liquid chromatography-tandem mass spectrometry analysis

Metabolites were quantified using a Waters Acquity UPLC system coupled to a Xevo TQD triple-quadrupole mass spectrometer (Waters). Chromatographic separation was achieved on an Acquity UPLC BEH C18 column (2.1 × 100 mm, 1.7 *μ*m) at 40°C. The mobile phase consisted of water with 0.1% formic acid (A) and methanol (B) with an 8-minute gradient as follows: 2 minutes at 95% A, a linear increase to 95% B over 4 minutes, a 1-minute hold at 95% B, and a 1-minute re-equilibration with 95% A. The flow rate was 0.3–0.4 mL/min. Detection was performed in positive electrospray ionization mode using Multiple Reaction Monitoring (Supplemental Table 2). Capillary voltage, cone voltage, and collision energy were set at 0.6 kV, 20 V, and 15 eV, respectively.

### 2.4 Determination of ${IC}_{50}$ and $K_{i}$ values

The percent activity for a given reaction was calculated by comparing metabolite formation between inhibitor-treated incubations and vehicle controls, as detailed below.

$$\%\;\text{Activity}=\frac{\text{Peak}\;\text{area}\;\text{of}\;\text{metabolite}\;\text{with}\;\text{inhibitor}}{\text{Peak}\;\text{area}\;\text{of}\;\text{metabolite}\;\text{with}\;\text{vehicle}}\times 100\%$$

To improve cannabinoid solubility without significantly impacting enzyme activity, 3% methanol (MeOH) was used as the vehicle for *Δ*8-THC, 11-OH-*Δ*8-THC, and *Δ*8-THC-COOH in all incubations. Initial screening studies were performed using recombinant P450-overexpressing microsomes and 1 or 10 *μ*M cannabinoid concentrations and validated using HLM. Cannabinoids that reduced activity by ≥ 50% at 1 *μ*M or 10 *μ*M in reactions containing either recombinant enzyme or HLM were further evaluated for ${\text{IC}}_{50}$ in recombinant P450-overexpressing microsomes as well as HLM. ${\text{IC}}_{50}$ assays were conducted across inhibitor concentrations of 0.1 – 100 *μ*M under consistent incubation conditions. All determinations were performed in triplicate for reproducibility. ${\text{IC}}_{50}$ values, defined as the concentration reducing enzyme activity by 50%, were calculated by nonlinear regression (see below) using GraphPad Prism 10.0 (GraphPad):

$$\%\;\text{Activity}=\text{Bottom}+\frac{\text{Top}-\text{Bottom}}{1+10^{\left(\mathrm{Log}\left[I\right]-\mathrm{Log}\left[{\text{IC}}_{50}\right]\right)}}$$

where Log[I] represents the logarithm of inhibitor concentration, Top is the highest % activity (set to 100%, assuming no inhibition without inhibitor), and Bottom is the lowest % activity (set to 0%, assuming full inhibition at high concentrations).

### 2.5 ${IC}_{50}$ shift assay

To characterize inhibition type, ${\text{IC}}_{50}$ shift studies were performed using recombinant P450-overexpressing microsomes. Assays were preincubated at 37°C for 30 minutes with cannabinoid with or without NADPH in the absence of a probe substrate. After preincubation, substrate was added and incubated for 30 minutes. ${\text{IC}}_{50}$ values were calculated as mentioned above using GraphPad Prism 10.0.

$${\text{IC}}_{50}\;\text{shift}=\frac{{\text{IC}}_{50}\;\text{with}\;30-\min\text{preincubation}\;\text{minus}\;\text{NADPH}}{{\text{IC}}_{50}\;\text{with}\;30-\min\text{preincubation}\;\text{plus}\;\text{NADPH}}$$

According to Food and Drug Administration guidance, a ${\text{IC}}_{50}$ shift > 1.5 indicates time-dependent inhibition.^11^

To determine *K*_i_ values for each P450 enzyme, substrate and inhibitor concentrations were selected based on known *K*_m_ and ${\text{IC}}_{50}$ values. Substrate concentrations were 2.5, 10, 30, and 60 *μ*M for diclofenac^26^ and warfarin,^27^ and 1, 5, 10, and 25 *μ*M for midazolam^28^ for CYPs 2C9 and 3A4, respectively. Internal standards (deuterated metabolites) were used to normalize metabolite peak areas. Lineweaver–Burk plots ($\frac{1}{v}$ vs $\frac{1}{S}$) were used to characterize inhibition type. Based on their inhibition type, reversible inhibition data were fitted to multiple models using nonlinear regression in GraphPad Prism 10.0 to estimate $K_{i}$ values.

$$v=\frac{V_{\max}\times S}{\left(1+\frac{I}{K_{i}}\right)\times \left(K_{m}+S\right)}\;\text{for}\;\text{noncompetitive}\;\text{inhibition}$$

$$v=\frac{V_{\max}\times S}{K_{m}\times \left(1+\frac{I}{K_{i}}\right)+S}\;\text{for}\;\text{competitive}\;\text{inhibition}$$

where I is inhibitor concentration, $K_{i}$ is the inhibition constant, S is the substrate concentration, and $K_{m}$ the Michaelis-Menten constant. Reaction velocity (v) refers to the rate of metabolite formation, calculated by dividing the metabolite peak area by the internal standard peak area for a given reaction.

${\text{IC}}_{50}$ and $K_{i}$ values were corrected for nonspecific binding using the unbound fraction term ($f_{u,\text{inc}}$). The $f_{u,\text{inc}}$ of 0.051 in HLM and 0.043 in recombinant P450-overexpressing microsomes for *Δ*9-THC, and 0.094 in HLM and 0.078 in recombinant P450-overexpressing microsomes for 11-OH-*Δ*9-THC were used to estimate the corrected inhibitory potency for *Δ*8-THC and 11-OH-*Δ*8-THC, respectively.^13^

$${\text{IC}}_{50,u}=f_{u,\text{inc}}\times {\text{IC}}_{50}$$

$$K_{i,u}=f_{u,\text{inc}}\times K_{i}$$

with ${\text{IC}}_{50,u}$ and $K_{i,u}$ the mean unbound ${\text{IC}}_{50}$ and $K_{i}$, respectively.

### 2.6 Prediction of in vivo DDI by static modeling

To predict the risk of clinical DDI, we applied static models of reversible inhibition.^29^ The area under the concentration-time curve ratio (AUCR) was used to estimate the impact of *Δ*8-THC and 11-OH-*Δ*8-THC on probe substrates.

For oral administration, the AUCR was calculated using eq.1, with A the effect of reversible inhibition, and B and C denoting time-dependent inhibition and induction respectively. Subscripts “h” and “g” denote hepatic and gut, respectively. B and C were set to 1 (not applicable here, because no time-dependent inhibition was observed). $F_{g}$ is the fraction of substrate available after intestinal metabolism, and it was set to 0.64 for diclofenac,^14^ 0.99 for warfarin,^30^ and 0.51 for midazolam.^31^ $f_{m}$ is the fraction of hepatic clearance of the substrate mediated by the P450 enzyme that is subject to inhibition/induction, which was set to 0.98 for diclofenac,^14^ 0.91 for warfarin,^32^ and 0.93 for midazolam.^31^ An AUCR≥1.25 suggests a significant presystemic hepatic DDI after oral administration.

(1) $$\text{AUCR}=\left(\frac{1}{\left[A_{g}\times B_{g}\times C_{g}\right]\times \left(1-F_{g}\right)+F_{g}}\right)\times \left(\frac{1}{\left[A_{h}\times B_{h}\times C_{h}\right]\times f_{m}+\left(1-f_{m}\right)}\right)$$

The $A_{g}$ was calculated using the $I_{g}$ (the inhibitor drug concentration in the gut) and $K_{i,u}$ values determined in recombinant P450-overexpressing microsomes (eq. 2), with the $I_{g}$ calculated as indicated in eq.3, with $F_{a}$ the fraction of absorbed inhibitor (*Δ*8-THC or 11-OH-*Δ*8-THC) set to 1.0; $K_{a}$, the intestinal absorption rate of inhibitor (*Δ*8-THC or 11-OH-*Δ*8-THC) set to 0.1 min^-1^; and $Q_{\text{en}}$, _the enterocyte_ blood flow set to 0.3 L/min.^29^

(2) $$A_{g}=\frac{1}{1+\frac{I_{g}}{K_{i}}}$$

(3) $$I_{g}=\frac{F_{a}\times K_{a}\times \text{Dose}}{Q_{\text{en}}}$$

The $A_{h}$ was calculated using the $I_{h}$ (the inhibitor drug concentration in the liver) and $K_{i,u}$ values determined in recombinant P450-overexpressing microsomes (see Results section; eq. 4), with the unbound maximum hepatic inlet concentration ($I_{h}$) calculated as indicated in eq.5. Due to the chemical similarities between *Δ*8-THC and *Δ*9-THC, the $f_{u,p}$ (the unbound fraction of drug in plasma) was set at 0.03 for both *Δ*8-THC and 11-OH-*Δ*8-THC.^33^ $R_{B}$, the ratio of drug concentration in blood ($C_{B}$) to drug concentration in plasma, was set to 0.40, and $Q_{\text{hep}}$, _the_ hepatic blood flow, was set to 1.5 L/min.^29^ $C_{\max}$ is the maximum total plasma concentration of inhibitor (*Δ*8-THC or 11-OH-*Δ*8-THC).^34^^,^^35^

(4) $$A_{h}=\frac{1}{1+\frac{I_{h}}{K_{i}}}$$

(5) Ih=[fu,p×(Cmax+Fa×Ka×DoseQhepRB)]

For inhalation exposure, ${\text{AUCR}}_{\text{sys}}$ was calculated using eqs. 6 and 7*,* with the $I_{\text{sys}}$ equaling the $C_{\max,u}$ which is the unbound peak plasma concentration. An ${\text{AUCR}}_{\text{sys}}\geq 1.02$ indicates a significant systemic DDI after inhalation exposure.^29^

(6) $${\text{AUCR}}_{\text{sys}}=1+\frac{I_{\text{sys}}}{K_{i,u}}$$

(7) $$I_{\text{sys}}=C_{\max,u}=f_{u,p}\times C_{\max}$$

## 3 Results

**Fig. 1:**
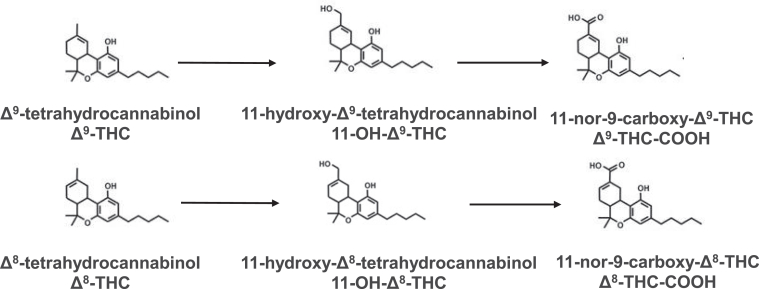
Chemical structures of *Δ*9-THC, *Δ*8-THC, and their metabolites.

**Fig. 2:**
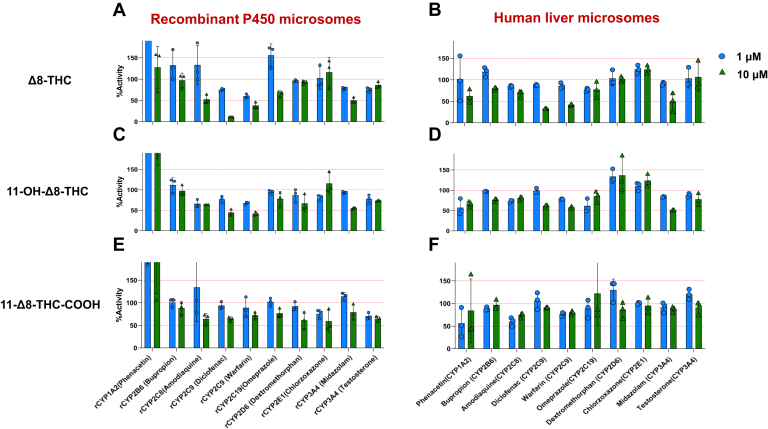
Inhibition of major hepatic P450 enzymes by *Δ*8-THC and its metabolites in recombinant P450 microsomes and HLMs. Panels A–E show the effects of 1 *μ*M (blue) and 10 *μ*M (green) *Δ*8-THC, 11-OH-*Δ*8-THC, and *Δ*8-THC-COOH. Data represent means from 3 independent experiments. Red dashed lines indicate 100% (no inhibition) and 50% activity thresholds. “r” refers to recombinant P450 microsomes.

The chemical structures of *Δ*9-THC, *Δ*8-THC, and their metabolites are shown in Fig. 1. Initial studies were performed to screen for possible cytochrome P450 inhibition by *Δ*8-THC, 11-OH-*Δ*8-THC, and *Δ*8-THC-COOH. Both *Δ*8-THC and 11-OH-*Δ*8-THC exhibited consistent inhibitory effects on both CYP2C9 and CYP3A4 in recombinant P450-overexpressing microsomes as well as HLM using P450-specific probe substrates. At 10 *μ*M, both cannabinoids reduced the formation of 4′-hydroxydiclofenac and 7′-hydroxywarfarin (CYP2C9 substrates) as well as 1′-hydroxymidazolam (CYP3A4 substrate) by > 40% (Fig. 2, A–D). In contrast, *Δ*8-THC-COOH exhibited minimal or no inhibition against any P450 enzymes tested (Fig. 2, E and F).

**Fig. 3:**
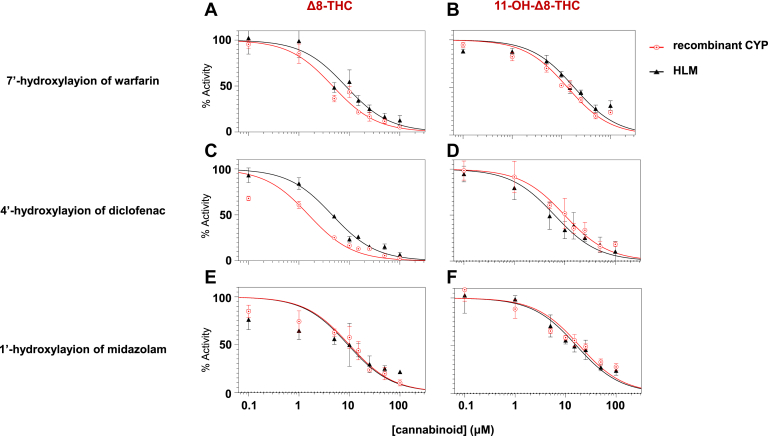
IC_50_ curves for the inhibition of CYP2C9 and CYP3A4 activity by *Δ*8-THC and 11-OH-*Δ*8-THC. Panels A–F show the concentration-dependent inhibition of the 4′-hydroxylation of diclofenac, 7′-hydroxylation of warfarin, and 1′-hydroxylation of midazolam in recombinant P450-overexpressing microsomes (red circles) and HLM (black triangles). The left panels show inhibition by *Δ*8-THC, and the right panels by 11-OH-*Δ*8-THC. Y-axis shows relative enzyme activity as compared to control reactions without cannabinoid. Rec, recombinant.

${\text{IC}}_{50}$ curves for *Δ*8-THC and 11-OH-*Δ*8-THC inhibition of P450 enzyme activity are shown in Fig. 3. Both compounds demonstrated concentration-dependent inhibition of CYPs 2C9 and 3A4 as indicated by reduced formation of 4′-hydroxydiclofenac (Fig. 3, A and B), 7′-hydroxywarfarin (Fig. 3. C and D), and 1′-hydroxymidazolam (Fig. 3, E and F) in both recombinant P450-overexpressing microsomes and HLM. The unbound ${\text{IC}}_{50}$ (${\text{IC}}_{50,u}$) values for *Δ*8-THC were < 0.5 *μ*M against CYPs 2C9 and 3A4 for both recombinant enzyme microsomes and HLM (Table 1), with the strongest inhibition observed for CYP2C9 in recombinant microsomes (${\text{IC}}_{50,u}$ = 0.07 ± 0.01 *μ*M for the 4′-hydroxylation of diclofenac, and 0.20 ± 0.03 *μ*M for the 7′-hydroxylation of warfarin). Similar inhibition was observed in HLM, with corresponding ${\text{IC}}_{50,u}$ values of 0.23 ± 0.03 *μ*M and 0.44 ± 0.14 *μ*M, respectively. Strong inhibition of CYP3A4 was also observed for *Δ*8-THC against 1′-hydroxyl-midazolam formation (${\text{IC}}_{50,u}$ = 0.44 ± 0.14 *μ*M for recombinant CYP3A4-overexpressing microsomes and 0.48 ± 0.11 *μ*M for HLM).

**Table 1:** Unbound ${\text{IC}}_{50}$ values for *Δ*^8^-THC and 11-OH-*Δ*^8^-THC against CYP2C9 and CYP3A4

Cannabinoid	Substrate	Metabolite Formed	Microsomes	${\text{IC}}_{50,u}$^a^, ^b^ (*μ*M)
*Δ*8-THC	Diclofenac	4′-Hydroxydiclofenac	rec CYP2C9	0.07 ± 0.01
			HLM	0.23 ± 0.03
	Warfarin	7′-Hydroxywarfarin	rec CYP2C9	0.20 ± 0.03
			HLM	0.44 ± 0.14
	Midazolam	1′-Hydroxymidazolam	rec CYP3A4	0.44 ± 0.14
			HLM	0.48 ± 0.11
11-OH- *Δ*8-THC	Diclofenac	4′-Hydroxydiclofenac	rec CYP2C9	0.79 ± 0.29
			HLM	0.71 ± 0.43
	Warfarin	7′-Hydroxywarfarin	rec CYP2C9	1.05 ± 0.13
			HLM	1.72 ± 0.10
	Midazolam	1′-Hydroxylmidazolam	rec CYP3A4	1.48 ± 0.21
			HLM	1.57 ± 0.32

rec, recombinant.

a ${\text{IC}}_{50,u}$ refers to the unbound ${\text{IC}}_{50}$ value, adjusted using the unbound fractions of *Δ*9-THC (0.051 in HLM, 0.043 in recombinant CYP450 microsomes) for *Δ*8-THC, and 11-OH-*Δ*9-THC (0.094 in HLM, 0.078 in recombinant CYP450 microsomes) for 11-OH-*Δ*8-THC.

b All values are expressed as the mean ± SD from 3 independent experiments.

11-OH-*Δ*8-THC exhibited moderate-strong inhibition across all P450 enzyme-substrate pairs, with ${\text{IC}}_{50,u}$ values ranged from 0.71 to 1.70 *μ*M in HLM and 0.79 to 1.50 *μ*M in recombinant P450-overexpressing microsomes. The strongest inhibition was observed against diclofenac 4′-hydroxylation formation in recombinant CYP2C9 microsomes with an ${\text{IC}}_{50,u}$ = 0.79 ± 0.29 *μ*M, which was approximately 11-fold higher than that of *Δ*8-THC.

**Fig. 4:**
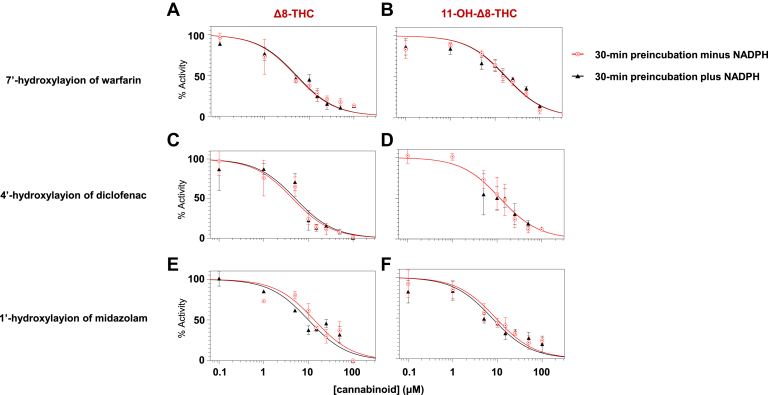
Reversible cytochrome P450 inhibition by *Δ*8-THC and 11-OH-*Δ*8-THC. Panels A–F show representative IC_50_ curves following a 30-minute preincubation with (black triangles) or without (red circles) NADPH for the 7′-hydroxylation of warfarin (CYP2C9), 4′-hydroxylation of diclofenac (CYP2C9), and the 1′-hydroxylation of midazolam (CYP3A4). The left panels show inhibition by *Δ*8-THC, and the right panels by 11-OH-*Δ*8-THC. Similar curves indicate reversible inhibition. Y-axis shows relative enzyme activity as compared with control reactions without cannabinoid.

To assess whether *Δ*8-THC and 11-OH-*Δ*8-THC exhibit time- and NADPH-dependent inhibition (hallmarks of irreversible or mechanism-based inhibition), ${IC}_{50}$ shift assays were conducted. Inhibitory potency was measured after a 30-minute preincubation in recombinant CYPs 2C9 and 3A4 microsomes with and without NADPH in the absence of substrate, respectively. ${IC}_{50}$ curves for *Δ*8-THC (Fig. 4, A, C, and E) and 11-OH-*Δ*^8^-THC (Fig. 4, B, D, and F) across CYPs 2C9- and 3A4-mediated reactions after 30-minute preincubation with or without NADPH. For both cannabinoids, no appreciable differences were observed between the +NADPH and –NADPH conditions. Table 2 summarizes the ${IC}_{50}$ shift values of *Δ*8-THC and 11-OH-*Δ*8-THC in CYPs 2C9- and 3A4-overexpressing microsomes, with the ${IC}_{50}$ shift reflecting the fold-change in inhibitory potency following a 30-minute preincubation with versus without NADPH. All of the ${IC}_{50}$ shift values observed in this study were below the ${IC}_{50}$ shift threshold of 1.5 that is generally used to distinguish between reversible and time-dependent inhibition.^11^ This suggests that neither *Δ*8-THC nor 11-OH-*Δ*8-THC undergoes time-dependent inhibition.

**Table 2:** The ${\text{IC}}_{50}$ shift of *Δ*8-THC and 11-OH-*Δ*8-THC in recombinant P450-overexpressing microsomes

Cannabinoid	Reaction	Microsomes	${\text{IC}}_{50}$ Shift^a^^,^^b^
*Δ*8-THC	4′-Hydroxylation of diclofenac	rec CYP2C9	1.24 ± 0.34
	7′-Hydroxylation of warfarin	rec CYP2C9	0.93 ± 0.02
	1′-Hydroxylation of midazolam	rec CYP3A4	0.85 ± 0.09
11-OH- *Δ*8-THC	4′-Hydroxylation of diclofenac	rec CYP2C9	0.95 ± 0.01
	7′-Hydroxylation of warfarin	rec CYP2C9	1.11 ± 0.08
	1′-Hydroxylation of midazolam	rec CYP3A4	0.83 ± 0.11

rec, recombinant.

a Values are expressed as the mean ± SD from 3 independent experiments, each assessing the inhibition of a P450 enzyme by a given cannabinoid.

b The ${\text{IC}}_{50}$ shift represents the fold-change between ${\text{IC}}_{50}$ values obtained with a 30-minute preincubation with NADPH versus without NADPH, as described in Material and methods.

**Fig. 5:**
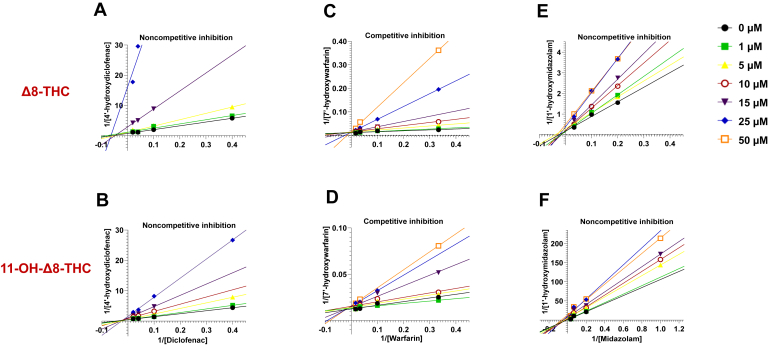
Representative Lineweaver–Burk plots for the inhibition of CYPs 2C9 and 3A4 in HLM by *Δ*8-THC (A, C, and E) and 11-OH-*Δ*8-THC (B, D, and F). Each panel displays double reciprocal plots of metabolite formation rate (1/V) versus substrate concentration (1/[S]) across a range of inhibitor concentrations (0 – 50 *μ*M). Panels A and B depict noncompetitive inhibition of diclofenac 4′-hydroxylation, panels C and D show competitive inhibition of warfarin 7′-hydroxylation, panels E and F demonstrate noncompetitive inhibition of midazolam 1′-hydroxylation.

To further characterize the reversible inhibition profile of *Δ*8-THC and 11-OH-*Δ*8-THC, both cannabinoids were evaluated over a concentration range of 0 – 50 *μ*M in incubations with recombinant P450 microsomes and HLM. Diclofenac and warfarin were used as probe substrates for CYP2C9, and midazolam for CYP3A4, with substrate concentrations ranging from 3 to 60 *μ*M (diclofenac, warfarin) and 1 – 25 *μ*M (midazolam) (see Material and methods for details). Consistent inhibition profiles were observed across both systems. Lineweaver–Burk plots were used to differentiate among inhibition mechanisms (Fig. 5). *Δ*8-THC and 11-OH-*Δ*8-THC exhibited noncompetitive inhibition of CYP2C9-mediated diclofenac metabolism and CYP3A4-mediated midazolam metabolism, indicated by shared X-axis intercepts. In contrast, competitive inhibition was observed for CYP2C9-mediated warfarin metabolism, as shown by shared Y-axis intercepts.

Unbound inhibition constants ($K_{i,u}$) were calculated using both recombinant P450-overexpressing microsomes and HLM. *Δ*8-THC demonstrated potent inhibition of CYP2C9, with $K_{i,u}$ values of 0.26 ± 0.01 *μ*M (recombinant CYP2C9) and 0.32 ± 0.06 *μ*M (HLM) for diclofenac 4′-hydroxylation, and even lower $K_{i,u}$ values observed for warfarin metabolism (0.16 ± 0.04 *μ*M and 0.23 ± 0.09 *μ*M for recombinant CYP2C9 and HLM, respectively; Table 3). CYP3A4 inhibition was also strong, with a $K_{i,u}$ of 0.52 ± 0.02 *μ*M. 11-OH-*Δ*8-THC exhibited generally weaker inhibition across all reactions, with $K_{i,u}$ values ranging from 0.40 ± 0.08 *μ*M to 1.20 ± 0.07 *μ*M. These data indicate that *Δ*8-THC may pose a significant drug interaction risk with CYP2C9 substrates such as warfarin and diclofenac and possibly with CYP3A4 substrates like midazolam.

**Table 3:** The unbound $K_{i}$ of *Δ*8-THC and 11-OH-*Δ*8-THC against CYPs 2C9 and 3A4

Cannabinoid	Reaction	Microsomes	$K_{i,u}$^a^, ^b^ (*μ*M)	Inhibition Type^c^
*Δ*8-THC	4′-Hydroxylation of diclofenac	rec CYP2C9 HLM	0.26 ± 0.01 0.32 ± 0.06	N
	7′-Hydroxylation of warfarin	rec CYP2C9 HLM	0.09 ± 0.04 0.23 ± 0.09	C
	1′-Hydroxylation of midazolam	rec CYP3A4 HLM	0.86 ± 0.28 0.52 ± 0.02	N
11-OH- *Δ*8-THC	4′-Hydroxylation of diclofenac	rec CYP2C9 HLM	0.73 ± 0.04 0.77 ± 0.04	N
	7′-Hydroxylation of warfarin	rec CYP2C9 HLM	0.40 ± 0.16 0.40 ± 0.08	C
	1′-Hydroxylation of midazolam	rec CYP3A4 HLM	0.89 ± 0.20 1.20 ± 0.07	N

rec, recombinant.

a $K_{i,u}$ refers to the unbound $K_{i}$ value, adjusted using the unbound fractions of *Δ*9-THC (0.051 in HLM, 0.043 in recombinant CYP450 microsomes) for *Δ*8-THC, and 11-OH-*Δ*9-THC (0.094 in HLM, 0.078 in recombinant CYP450 microsomes) for 11-OH-*Δ*8-THC.

b Values are expressed as mean ± SD from 3 independent experiments evaluating the inhibition of each P450 reaction by each inhibitor.

c N, noncompetitive inhibition; C, competitive inhibition.

To evaluate the potential clinical impact of *Δ*8-THC on DDI, static models were used to predict the AUCR.^29^ Due to their structural and pharmacokinetic similarities, *Δ*8-THC and 11-OH-*Δ*8-THC were assumed to share equivalent $C_{\max}$ values with *Δ*9-THC and 11-OH-*Δ*9-THC at matched inhalation doses, respectively.^14^^,^^36^

$K_{i,u}$ values were used to calculate AUCR, and for orally administered agents, a predicted AUCR ≥ 1.25 indicates potential for significant presystemic DDIs. In the present model, DDI risk increased in a dose-dependent manner (Table 4). At a low *Δ*8-THC dose (10 mg), predicted AUCRs were 2.29 (diclofenac), 1.61 (warfarin), and 2.34 (midazolam), whereas at a medium *Δ*8-THC dose (20 mg), AUCRs rose to 3.04 (diclofenac), 2.14 (warfarin), and 2.92 (midazolam), respectively. At a relatively high dose (40 mg), AUCRs further increased to 4.48 (diclofenac), 3.05 (warfarin), and 3.94 (midazolam). For 11-OH-*Δ*8-THC, predicted AUCRs were comparatively lower than that of *Δ*8-THC. They were 1.79 (diclofenac), 1.35 (warfarin), and 1.89 (midazolam) at a low *Δ*8-THC dose (10 mg), 2.12 (diclofenac), 1.66 (warfarin), and 2.23 (midazolam) at a medium *Δ*8-THC dose (20 mg), and 2.72 (diclofenac), 2.23 (warfarin), and 2.73 (midazolam) at a relatively high *Δ*8-THC dose (40 mg).

**Table 4:** The predicted area-under-the-curve ratio (AUCR) for P450-mediated *Δ*8-THC drug interactions using static models

Cannabinoid	Route of Administration	Dose (mg)	$C_{\max}$^a^ (*μ*M)	Predicted AUCR^b^^c^		
				CYP2C9		CYP3A4
				Diclofenac	Warfarin	Midazolam
*Δ*8-THC	Oral	10	0.0079	**1.61**	**2.29**	**2.34**
		20	0.0178	**2.14**	**3.04**	**2.92**
		40	0.0480	**3.05**	**4.48**	**3.94**
	Inhalation	25	0.2500	**1.02**	**1.08**	1.01
		70	0.7000	**1.07**	**1.23**	**1.04**
		100	0.9900	**1.09**	**1.32**	**1.06**
11-OH-*Δ*8-THC	Oral	10	0.0150	**1.35**	**1.79**	**1.89**
		20	0.1000	**1.66**	**2.12**	**2.23**
		40	0.1200	**2.23**	**2.72**	**2.73**
	Inhalation	25	0.0120	1.00	1.00	1.00
		70	0.0240	1.00	1.00	1.00
		100	0.0360	1.00	1.00	1.00

a The inhalation doses and ${C_{\max}}^{\prime }s$ used for AUCR prediction are the inhalation doses and ${C_{\max}}^{\prime }s$ described for *Δ*9-THC.^14^^,^^34^^,^^36^ The oral doses and ${C_{\max}}^{\prime }s$ used for AUCR prediction are the oral doses and ${C_{\max}}^{\prime }s$ for *Δ*8-THC.^35^

b AUCR’s were estimated using the $K_{i}$ values in Table 3 (see Material and methods for details).

c Bolded numbers indicate a strong potential for DDI. If the predicted AUCR is greater than or equal to 1.25 or 1.02 for oral administration or inhalation exposure, respectively, a DDI is predicted.^29^

For inhalation exposure, a predicted AUCR ≥ 1.02 indicates potential for systemic DDI.^29^ At low (25 mg) and moderate (54 mg) *Δ*8-THC inhalation doses, AUCRs for midazolam remained near 1.00 (Table 4).^14^^,^^29^^,^^34^, ^35^, ^36^ At a high inhalation dose (70 mg), AUCRs increased slightly to 1.07 (diclofenac), 1.23 (warfarin), and 1.04 (midazolam). However, at a very high dose (100 mg), values reached 1.09 (diclofenac), 1.32 (warfarin), and 1.06 (midazolam). For 11-OH-*Δ*8-THC, AUCRs remained at 1.00 across all inhalation doses tested.

## 4 Discussion

This is the first study to investigate the potential inhibitory effects of *Δ*8-THC on hepatic P450-mediated drug metabolism. Results from this study showed that *Δ*8-THC and its active metabolite, 11-OH-*Δ*8-THC, inhibited the activities of CYPs 2C9 and 3A4 in both recombinant P450-overexpressing systems and in HLM. Minimal or no inhibition of enzyme activity was observed for other major hepatic P450 enzymes tested in this study. Interestingly, although this inhibition was observed for both substrates tested for CYP2C9 (diclofenac and warfarin), this inhibition appeared to be substrate specific for CYP3A4, with inhibition observed for midazolam but not testosterone. CYP3A4 is known for its large and flexible active site that accommodates a wide variety of substrates. Both midazolam and testosterone bind in the same active site,^37^ but their binding orientations differ, with midazolam having a more flexible binding mode, whereas testosterone’s steroid structure results in a more rigid fit.^37^ Although midazolam binds near the heme group within the CYP3A4 molecule,^38^ interacting with key residues such as Phe108, Phe304, Ile120, and Ala305,^39^ the key interacting residues between testosterone and CYP3A4 are different and include Ser119, Ile301, Ala305, and Leu373.^40^

Interestingly, *Δ*8-THC and 11-OH-*Δ*8-THC were competitive inhibitors for CYP2C9-mediated 7′-hydroxylation of warfarin but noncompetitive inhibitors for 4′-hydrxolation of diclofenac suggesting that *Δ*8-THC and 11-OH-*Δ*8-THC share the same binding pocket with warfarin but not for diclofenac on the CYP2C9 protein. These differences in inhibition type could potentially be explained by the fact that both warfarin and diclofenac interact with hydrophobic sites within the CYP2C9 enzyme, but this interaction is with different amino acid residues.^41^ Further structural studies examining *Δ*8-THC binding within the CYP2C9 and CYP3A4 molecules will be necessary to better assess the structural mechanisms underlying these interactions.

Given that *Δ*8-THC shares high structural similarity with *Δ*9-THC, similar inhibition profiles were expected for major hepatic P450 enzymes. Previous in vitro studies showed that *Δ*9-THC inhibited the activities of multiple P450 enzymes including CYPs 1A2 ($K_{i,u}$ = 0.090 ± 0.027 *μ*M for recombinant CYP1A2 and 0.10 ± 0.056 *μ*M for HLM), 2B6 ($K_{i,u}$ = 0.25 ± 0.043 *μ*M for recombinant CYP2B6 and 0.38 ± 0.029 *μ*M for HLM), 2C9 ($K_{i,u}$ = 0.073 ± 0.023 *μ*M for recombinant CYP2C9 and 0.17 ± 0.046 *μ*M for HLM), 2C19 ($K_{i,u}$ = 0.056 ± 0.018 *μ*M for recombinant CYP2C19 and 0.21 ± 0.082 *μ*M for HLM), and 2D6 ($K_{i,u}$ = 0.11 ± 0.015 *μ*M for recombinant CYP2D6 and 0.28 ± 0.030 *μ*M for HLM).^13^ Similarly, the active *Δ*9-THC metabolite, 11-OH-*Δ*9-THC, also inhibited CYPs 2B6 ($K_{i,u}$ = 0.086 ± 0.066 *μ*M for recombinant CYP2B6 and 0.26 ± 0.041 *μ*M for HLM), 2C9 ($K_{i,u}$ = 0.057 ± 0.044 *μ*M for recombinant CYP2C9 and 0.21 ± 0.032 *μ*M for HLM), and 2D6 ($K_{i,u}$ = 0.15 ± 0.067 *μ*M for recombinant CYP2D6 and 0.32 ± 0.24 *μ*M for HLM).^13^ In contrast, *Δ*8-THC and 11-OH-*Δ*8-THC exhibited a relatively narrow profile of cytochrome P450 inhibition, targeting 2 major hepatic enzymes, CYPs 2C9 and 3A4. Similar to that observed previously for *Δ*9-THC-COOH,^13^ *Δ*8-THC-COOH exhibited no inhibition of any major hepatic P450 enzymes. It is important to note that both *Δ*8-THC and 11-OH-*Δ*8-THC exhibited an inhibitory effect on CYP3A4-mediated 1′-hydroxylation of midazolam—a result not observed previously with *Δ*9-THC and 11-OH-*Δ*9-THC.^13^ In addition, *Δ*8-THC and *Δ*9-THC differ in their mechanisms of inhibition. *Δ*9-THC and 11-OH-*Δ*9-THC act as competitive inhibitors of CYP2C9-mediated 4′-hydroxylation of diclofenac, whereas *Δ*8-THC and 11-OH-*Δ*8-THC inhibit the same pathway noncompetitively. Variations in the inhibition profiles and mechanisms of *Δ*8-THC and *Δ*9-THC imply that the slight structural shift between them—namely, the position of the double bond in the cyclohexene ring—may alter their binding affinity for P450 enzymes, and further identification of the key *Δ*8-THC-enzyme interaction sites will be essential to understanding its inhibitory effects and metabolic behavior.

Diclofenac is a commonly used nonsteroidal anti-inflammatory drug, metabolized primarily by CYP2C9.^42^ Previous in vitro studies suggested that the inhibition of CYP2C9-mediated 4′-hydroxylation of diclofenac by *Δ*9-THC and 11-OH-*Δ*9-THC can reduce diclofenac’s clearance, leading to ≥ 44% increase in systematic exposure after a 40 mg oral dose of *Δ*9-THC based on mechanistic static modeling.^13^ Similar $K_{i}$ data were observed for *Δ*8-THC and 11-OH-*Δ*8-THC in the present study as were observed for *Δ*9-THC and 11-OH-*Δ*9-THC in these previous studies. Static modeling performed in the present study predicted strong DDI risks with oral *Δ*8-THC for diclofenac; inhaled *Δ*8-THC posed minimal risk at typical usage levels. The inhibition of CYP2C9-mediated 4′-hydroxylation of diclofenac observed with *Δ*8-THC in the present study were predicted to resulting in a ≥ 119% increase in systematic exposure after a 10 mg oral dose of *Δ*8-THC, an effect that becomes more dramatic at higher *Δ*8-THC doses including a 348% increase after a 40 mg oral dose of *Δ*8-THC. Another study suggested a > 560% increase in systematic exposure after a 130 mg oral dose of *Δ*9-THC based on mechanistic static modeling,^43^ which is similar to our results. Elevated plasma levels of diclofenac might induce side effects such as gastrointestinal bleeding, renal impairment, and cardiovascular issues.^44^

Warfarin is a commonly prescribed anticoagulant and, like diclofenac, is also predominantly metabolized by CYP2C9.^45^ Previous in vitro studies indicated that both *Δ*9-THC and 11-OH-*Δ*9-THC can inhibit CYP2C9-mediated 7′-hydroxylation of warfarin, potentially increasing systemic exposure and elevating the risk of adverse effects.^46^ Similar inhibitory potency ($K_{i}$ values) was observed in the present study for *Δ*8-THC and 11-OH-*Δ*8-THC, consistent with findings for *Δ*9-THC and its metabolite, 11-OH-*Δ*9-THC. Static modeling performed in the current study predicted a strong DDI risk between warfarin and orally administered *Δ*8-THC. The predicted AUCR suggests at least a 61% increase in systemic warfarin exposure after a 10 mg oral dose of *Δ*8-THC, with even greater increases at higher doses including a 205% increase after 40 mg oral dose of *Δ*8-THC. Given warfarin’s narrow therapeutic window, elevated plasma levels may lead to serious adverse outcomes such as excessive bleeding or, paradoxically, clotting due to unstable anticoagulation.^47^ Clinical case reports have documented increased bleeding risks in patients co-using cannabis products, further supporting the predicted interaction between *Δ*8-THC and warfarin.^48^

Midazolam is a widely used benzodiazepine for sedation and is primarily metabolized by CYP3A4.^39^ Previous in vitro studies reported that CBD inhibits CYP3A4-mediated 1′-hydroxylation of midazolam ($K_{i,u}$ = 0.093 ± 0.037 *μ*M for recombinant CYP3A4 and 0.22 ± 0.044 *μ*M for HLM), resulting in a > 8-fold increase in systemic exposure following an 800 mg oral dose of CBD, as predicted by mechanistic static modeling.^13^ Although *Δ*9-THC does not inhibit CYP3A4, the present study found that both *Δ*8-THC and 11-OH-*Δ*8-THC inhibit CYP3A4-mediated 1′-hydroxylation of midazolam. Static modeling predicted at least a 134% increase in systemic midazolam exposure after a 10 mg oral dose of *Δ*8-THC, with higher doses leading to more pronounced effects including a 294% increase after a 40 mg oral dose of *Δ*8-THC. Accumulation of midazolam may result in prolonged sedation and increased risk of respiratory depression. Clinical studies have shown that cannabis use is associated with heightened sedation and greater need for adjunct sedatives, supporting the potential for a clinically significant *Δ*8-THC–midazolam interaction.^49^

There were several limitations with the present study. Unlike *Δ*9-THC, information about the disposition and pharmacokinetic profile of *Δ*8-THC is extremely limited. To predict DDIs, $C_{\max}$ values for *Δ*9-THC inhalation were used in the *Δ*8-THC inhalation model, which may cause inaccuracies in predictions for its clinical impact. Although previous studies suggest that *Δ*8-THC and *Δ*9-THC have similar metabolism pathways and pharmacokinetic profiles, *Δ*8-THC is metabolized slower in the liver.^50^ One clinical study found that *Δ*8-THC has a higher inhalation $C_{\max}$ than that of *Δ*9-THC.^35^^,^^51^ Therefore, the AUCRs for inhalation dose calculated in the present study may be underestimating the impact of *Δ*8-THC inhalation for a given DDI.

Static models for drug-metabolizing enzymes reflect worst-case scenario predictions assuming constant enzyme activity and drug exposure. Predictions using physiologically based pharmacokinetic models, which integrate multiple dynamic processes, such as drug transporter-mediated enterohepatic uptake, renal clearance,^52^ drug transporter-mediated intestinal absorption,^53^^,^^54^ and intestinal metabolism^55^ within a single cohesive system, should be developed to improve predictions for DDI between *Δ*8-THC and agents metabolized by CYPs 3A4 and 2C9. In addition to DDI predictions in healthy subjects, the ability to predict DDI in pediatric,^56^ geriatric,^57^ pregnancy,^58^ and other special populations is a promising advantage for using physiologically based pharmacokinetic modeling over static DDI models.

Taken together, this study provides the first compelling in vitro evidence that *Δ*8-THC and its active metabolite, 11-OH-*Δ*8-THC, inhibit the key hepatic enzymes CYP2C9 and CYP3A4, indicating a significant potential for DDI with widely used medications such as diclofenac, warfarin, and midazolam.

## Conflict of interest

The authors declare no conflicts of interest. Dr Shelby Coates is currently employed by Pfizer; however, her employment had no influence on the design, conduct, or reporting of this study. The remaining authors have no financial or personal relationships with commercial entities that could have influenced the work presented in this manuscript.
