# Supplementary material for: Inhibitory effects of Δ8-tetrahydrocannabinol on major hepatic cytochrome P450 enzymes and implications for drug disposition 🅂
Source: Drug Metab Dispos. Author manuscript; Available in PMC 2025 Dec 4. (PMC12597552; doi:10.1016/j.dmd.2025.100122)
Supplement: Supplementary Materials Final [file NIHMS2109622-supplement-Supplementary_Materials_Final.pdf]

Supplemental Tables

Table S1. Incubation conditions for assays with recombinant P450-overexpressing microsomes.

CYP Enzyme	Substrate	[Substrate] (μM)[a]	Incubation Time (min)	References
3A4	Midazolam	5	15	[53]
	Testosterone	25	30	[53]
2C9	Diclofenac	10	15	[54]
	Warfarin	10	30	[39]
2D6	Dextromethorphan	5	30	[55]
1A2	Phenacetin	10	30	[56]
2E1	Chlorzoxazone	100	15	[57]
2C8	Amodiaquine	2	15	[13]
2C19	Omeprazole	1	30	[58]
2B6	Bupropion	100	30	[59]

[a] The substrate concentrations used were similar to the known K_m for each enzyme against each corresponding substrate (references listed in the left column).

Table S2. Mass spectrometry conditions for substrate and metabolite measurements.

CYP	Substrate	RT[a] (min)	Q1>Q3	Metabolite formed	RT[a] (min)	Q1>Q3
3A4	midazolam	4.30	326>291	1-hydroxymidazolam	4.53	342.2>324
	testosterone	5.40	289>97	6ß-hydroxytestosterone	4.70	325>269
2C9	warfarin	5.30	309.2>163.3	7-hydroxywarfarin	5.10	325.3>179.2
	diclofenac	5.67	296>214	4-hydroxydiclofenac	5.22	312>266.1
2D6	dextromethorphan	4.30	272.3>147	dextrorphan	3.68	258.1>199
1A2	phenacetin	3.76	180.1>138	acetaminophen	2.06	152.06>110.16
2E1	chlorzoxazone	4.93	169.5>113	6-hydroxychlorzoxazone	3.99	185.56>130
2C8	amodiaquine	3.04	357.3>284.1	N-desethylamodiaquine	2.97	328>283
2C19	omeprazole	4.32	346>198	5-hydroxyomeprazole	4.05	362>214
2B6	bupropion	4.02	240>184	hydroxybupropion	3.91	256>139

[a] RT, reaction time.

Supplemental Figure S1. Expression of V5-tagged P450s in recombinant P450-overexpressing HEK293 microsomes. Western blow analysis was performed for 25 µg microsomal protein for each P450-overexpressing HEK293 cell line using anti-V5 and anti-calnexin antibodies. V5-HRP bands indicated the expression of each V5-tagged P450 enzyme while calnexin bands served as the loading control for microsomal protein samples. No V5 tag was detected in microsomes from parental HEK293 cells.
