# Supplementary figures and images for: Inhibitory effects of Δ8-tetrahydrocannabinol on major hepatic cytochrome P450 enzymes and implications for drug disposition 🅂
Source: Drug Metab Dispos. Author manuscript; Available in PMC 2025 Dec 4. (PMC12597552; doi:10.1016/j.dmd.2025.100122)

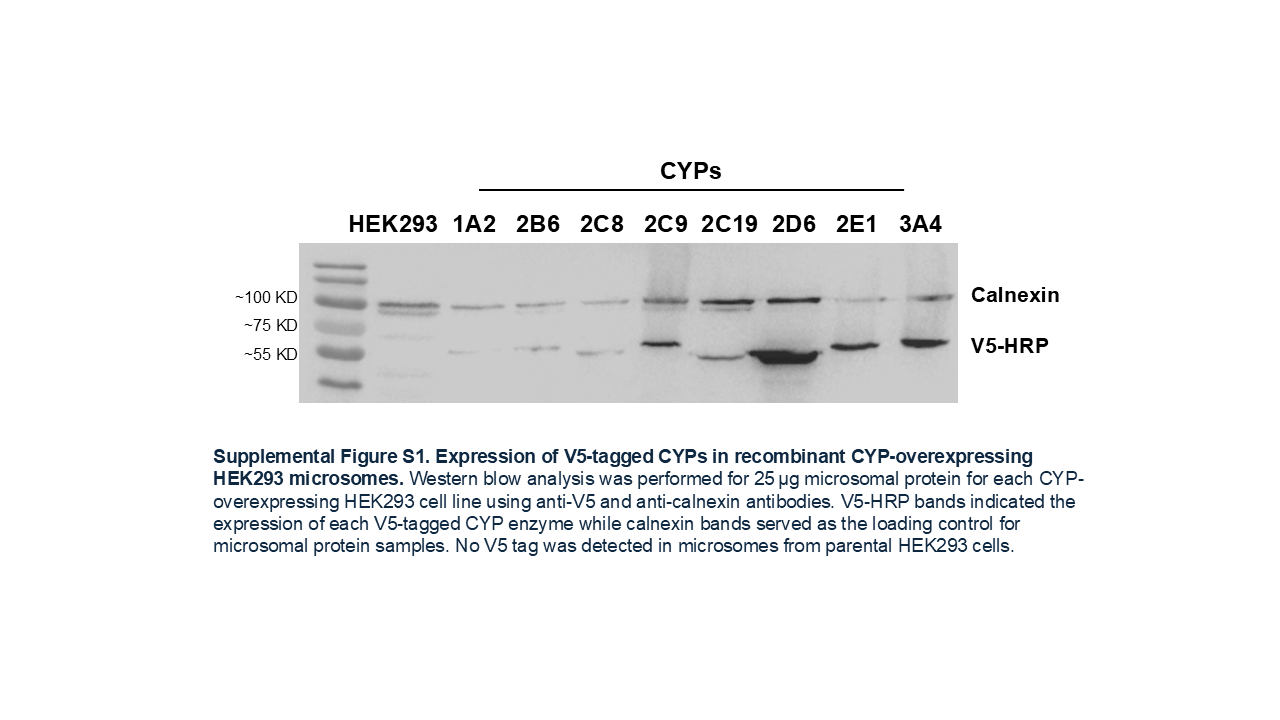

Supplement: Supplemental Figure 1 [file NIHMS2109622-supplement-Supplemental_Figure_1.tif]
