# Supplementary material for: Inhibitory effects of Δ8-tetrahydrocannabinol on major hepatic cytochrome P450 enzymes and implications for drug disposition
Source: Drug Metab Dispos. 2025 Jul 16;53(9):100122. doi: 10.1016/j.dmd.2025.100122 (PMC12597552; doi:10.1016/j.dmd.2025.100122)
Supplement: Supplementary file 1 [file mmc1.pdf]

Supplemental Tables

Supplemental Table 1. Incubation conditions for assays with recombinant P450 microsomes.

P450 enzymes	Substrate	[Substrate] (μM)[a]	Incubation Time (min)
CYP3A4	Midazolam	5	15
	Testosterone	25	30
CYP2C9	Diclofenac	10	15
	Warfarin	10	30
CYP2D6	Dextromethorphan	5	30
CYP1A2	Phenacetin	10	30
CYP2E1	Chlorzoxazone	100	15
CYP2C8	Amodiaquine	2	15
CYP2C19	Omeprazole	1	30
CYP2B6	Bupropion	100	30

[a] The substrate concentrations used were similar to the known K_m for each enzyme against each corresponding substrate (references listed in the left column).

Supplemental Table 2. Mass spectrometry conditions for substrate and metabolite measurements.

P450 enzymes	Substrate	RT[a] (min)	Q1>Q3	Metabolite formed	RT[a] (min)	Q1>Q3
CYP3A4	midazolam	4.30	326>291	1-hydroxymidazolam	4.53	342.2>324
	testosterone	5.40	289>97	6ß-hydroxytestosterone	4.70	325>269
CYP2C9	warfarin	5.30	309.2>163.3	7-hydroxywarfarin	5.10	325.3>179.2
	diclofenac	5.67	296>214	4-hydroxydiclofenac	5.22	312>266.1
CYP2D6	dextromethorphan	4.30	272.3>147	dextrorphan	3.68	258.1>199
CYP1A2	phenacetin	3.76	180.1>138	acetaminophen	2.06	152.06>110.16
CYP2E1	chlorzoxazone	4.93	169.5>113	6-hydroxychlorzoxazone	3.99	185.56>130
CYP2C8	amodiaquine	3.04	357.3>284.1	N-desethylamodiaquine	2.97	328>283
CYP2C19	omeprazole	4.32	346>198	5-hydroxyomeprazole	4.05	362>214
CYP2B6	bupropion	4.02	240>184	hydroxybupropion	3.91	256>139

[a] RT, reaction time.

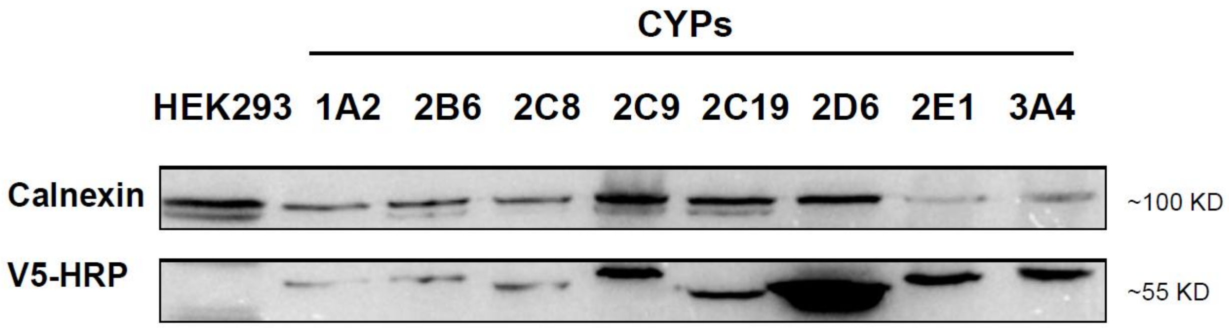

Supplemental Figure 1. Expression of V5-tagged P450 enzymes in recombinant P450-overexpressing HEK293 microsomes. Western blot analysis was performed for 25 μg microsomal protein for each P450-overexpressing HEK293 cell line using anti-V5 and anti-calnexin antibodies. V5-HRP bands indicated the expression of each V5-tagged CYP enzyme while calnexin bands served as the loading control for microsomal protein samples. No V5 tag was detected in microsomes from parental HEK293 cells.
